# LBNet: an optimized lightweight CNN for mammographic breast cancer classification with XAI-based interpretability

**DOI:** 10.1038/s41598-025-31642-6

**Published:** 2025-12-17

**Authors:** Jalal Ahmmed, Faruk Ahmed, Md Alamgir Kabir, Md Taimur Ahad, Mehreen Afsar Jadoon, Atiq ur Rehman, Amine Bermak

**Affiliations:** 1 Department of Computer Science and Engineering, Daffodil International University, Dhaka, Bangladesh; 2 Department of Software Engineering, Daffodil International University, Dhaka, Bangladesh Bangladesh; 3 Department of Management Information Systems, Independent University, Dhaka, Bangladesh; 4 College of Public Policy, Hamad Bin Khalifa University, Doha, Qatar; 5 College of Science and Engineering, Hamad Bin Khalifa University, Doha, Qatar

**Keywords:** Breast cancer, Mammography, Lightweight CNN, Explainable AI, SHAP, Grad-CAM, Transfer learning, Cancer, Computational biology and bioinformatics, Health care, Mathematics and computing

## Abstract

Breast cancer represents a major worldwide health burden, marked by high incidence and mortality rates across diverse socioeconomic populations. While deep learning has enabled advances in automated mammographic analysis, existing models often suffer from high computational complexity. They also face limited generalizability and a lack of interpretability. To overcome these challenges, this research introduces LBNet, a lightweight and interpretable convolutional neural network (CNN) built for accurate and efficient breast cancer detection, particularly in resource-constrained settings. With only 2.4 million trainable parameters, LBNet consists of five convolutional layers, leveraging ReLU activation, batch normalization, and max-pooling to optimize feature extraction while maintaining computational efficiency. LBNet was trained on the RSNA dataset using the Adam optimizer and five-fold cross-validation. It achieved 97.28% accuracy. For cancer cases, precision was 99% and recall was 96%. For non-cancer cases, precision was 96% and recall was 99%. In comparison, baseline models such as VGG19, SE-ResNet152, and ResNet152V2 yielded lower accuracies of 87.54%, 87.50%, and 85.24%, respectively, while transfer learning approaches peaked at 87.37% accuracy. LBNet’s generalizability was validated in external datasets, achieving 99.54% accuracy on CBIS-DDSM and 98.50% on MIAS. To enhance clinical trust, this work integrated SHAP (SHapley Additive exPlanations) and Grad-CAM (Gradient-weighted Class Activation Mapping). These methods effectively highlighted diagnostically relevant regions in mammograms. This improved prediction transparency. LBNet demonstrates strong potential as an accurate, efficient, and interpretable solution for breast cancer screening, and future studies could explore its extension to multi-view mammography and real-time clinical deployment.

## Introduction

**Fig. 1:**
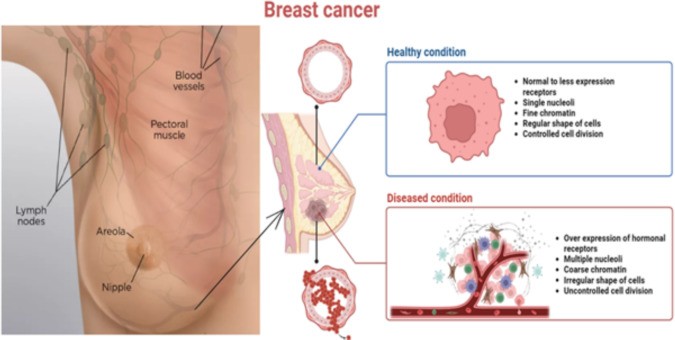
Breast cancer image (amended from).

Breast cancer remains one of the foremost global health concerns, significantly contributing to cancer incidence and mortality across both industrialized and developing nations^1^. According to the World Health Organization (WHO), approximately one in every ten newly diagnosed cancers worldwide is breast cancer, making it the most frequently diagnosed cancer among women^2^. Recent WHO and International Agency for Research on Cancer (IARC) reports indicate that around 2.3 million women were diagnosed with breast cancer in 2022. This led to nearly 670,000 deaths globally^3^. Moreover, IARC projects that by 2050, the global burden could rise to about 3.2 million new cases. It may also reach 1.1 million deaths annually if current trends continue^4^. In the United States alone, approximately 316,950 new invasive cases and 42,170 deaths are anticipated in 2025^5^. Therefore, early detection is vital, as breast cancer often manifests through symptoms such as palpable lumps or skin texture changes, which are primarily identified through imaging techniques like mammography^6^ (Fig. 1).

Traditional computer-aided diagnosis (CAD) systems, introduced in the 1990s, relied on hand-crafted features such as texture and shape, combined with classical classifiers like SVMs and decision trees^7^. These systems achieved moderate sensitivity in detecting masses and microcalcifications but suffered from high false-positive rates. The emergence of deep learning (DL) enabled end-to-end feature learning directly from raw mammograms, significantly improving detection accuracy^8^. Subsequent advancements incorporated transfer learning (TL), multi-view analysis (CC and MLO), and ensemble methods to further enhance performance. Despite these gains, deep CAD systems continue to face challenges in computational efficiency, interpretability, and generalization across diverse datasets and imaging protocols^9^. These limitations restrict clinical adoption, particularly in low-resource settings, underscoring the need for lightweight and transparent frameworks.

Recent advances in DL, particularly convolutional neural networks (CNNs), have transformed automated image-based cancer detection^10^. State-of-the-art architectures such as VGG19, DenseNet201, ResNet152V2, and InceptionV3 have demonstrated strong performance on large-scale mammographic datasets^11–13^. TL from pre-trained ImageNet models has further reduced training time and improved convergence on medical images^14,15^. However, practical deployment in clinical environments remains limited by the critical challenges outlined above^16^.

Many existing studies have developed DL models for medical image analysis, particularly using CNNs for mammographic breast cancer classification. Firstly, these models often suffer from high computational complexity, making them unsuitable for deployment in real-time or resource-limited environments. For example, Imandi et al.^17^ and Abbas et al.^18^ proposed high-performing models but did not address the issue of computational efficiency, which is critical for practical applications in embedded systems or point-of-care diagnostics. Secondly, although TL improves training efficiency, several studies report domain adaptation issues. Eva et al.^19^ found that models pre-trained on non-medical datasets perform poorly on mammographic images due to domain mismatch, highlighting the need for domain-specific adaptation. Thirdly, most DL models used in breast cancer classification act as “black boxes,” making it complicated for clinicians to fully comprehend or rely on the automated decisions. Although models proposed by Minh et al.^20^ and Marey et al.^21^ achieved high accuracy, they did not incorporate explainable mechanisms to provide insight into their decision-making processes, which limits their clinical applicability. Lastly, many models lack generalizability across diverse mammographic datasets. For example, Hou et al.^22^ found that performance often varies due to dataset-specific characteristics, such as differences in imaging equipment, class imbalance, and noise. These limitations underscore the need for a lightweight, explainable, and domain-adapted CNN. Such an architecture would address challenges in computational efficiency, domain adaptation, interpretability, and generalizability. It would enable effective and trustworthy mammographic classification.

To bridge these limitations, this research introduces a lightweight and interpretable DL framework for breast cancer detection using mammographic images. At its core is LBNet (*L*ightweight *B*reast *Net*work), a compact CNN model designed to deliver high classification accuracy while minimizing computational overhead. The proposed model is optimized for environments with limited hardware resources, without compromising classification performance. To address the critical issue of interpretability, this framework integrates post-hoc explainable AI (XAI)^23^ techniques, specifically, SHapley Additive exPlanations (SHAP)^24^ and Gradient-weighted Class Activation Mapping (Grad-CAM)^25^, which provide global and local interpretability of model predictions.

The main contributions and novelty of this work are as follows:

1. LBNet demonstrates high accuracy with extreme efficiency, achieving 97.28% accuracy using only 2.4M parameters. It outperforms six deep CNNs (VGG19, ResNet152V2, DenseNet201, etc.) trained from scratch or via transfer learning.
2. The model shows superior cross-dataset generalizability, validated on CBIS-DDSM (99.54%) and MIAS (98.50%), maintaining performance across diverse imaging conditions.
3. LBNet integrates XAI for enhanced interpretability. SHAP and Grad-CAM provide pixel-level and region-level explanations, enabling better understanding of model decisions.
4. The framework offers real-time, low-resource viability, with 23.3 ms/image inference, 7.92 GFLOPs, and 11.3 MB memory, making it suitable for point-of-care and low-resource settings.

This work is novel in unifying accuracy, efficiency, interpretability, and generalizability within a lightweight framework, making it a practical step toward scalable AI-assisted breast cancer screening.

## Related work

Deep Learning (DL) algorithms have significantly advanced breast cancer detection and analysis. Numerous studies have focused on optimizing DL models through various techniques, including Transfer Learning (TL), feature extraction, and classifier integration, to enhance performance.

In one group of studies, researchers investigated the application of large-scale datasets, localization-based architectures, and hybrid learning models for breast cancer detection. For instance, Martiniussen et al.^26^ conducted a retrospective study using 129,434 screening mammograms from BreastScreen Norway and evaluated two DL-based AI models for detection and localization. Both models achieved an AUC of 0.93, correctly identifying over 82% of screen-detected cancers at the lower threshold and more than 92% at the higher threshold. This large-scale evaluation demonstrated the robustness of DL-based systems in clinical screening. Similarly, Rahman et al.^27^ developed an advanced deep learning framework combining U-Net and YOLO architectures for simultaneous detection and localization of breast lesions in mammography images. Evaluated on the MIAS dataset, their model achieved 93% accuracy and an AUC of 98.6%, confirming the advantage of hybrid CNN-based approaches for precise lesion localization. Furthermore, Murty et al.^28^ proposed an integrative hybrid deep learning method by combining the Wisconsin Breast Cancer Database and the CBIS-DDSM dataset. Their CNN-based hybrid model, trained and fine-tuned across tabular and imaging data, achieved 96% accuracy and demonstrated improved adaptability across multiple breast imaging modalities. Again, both Laaffat et al.^29^ and Ruchai et al.^30^ explored the effectiveness of MobileNetV2 in breast cancer classification. Laaffat et al.^29^ demonstrated that fine-tuning MobileNetV2 significantly improved performance, achieving 97% accuracy, while Ruchai et al.^30^ emphasized the role of data augmentation in enhancing TL performance, reporting 95.8% accuracy. These studies highlight the importance of integrating large-scale datasets and localization-driven models. They also emphasize hybrid architectures, fine-tuning, and augmentation. These approaches enhance diagnostic accuracy and generalizability in breast cancer detection.

A second group of researchers has contributed to the field by incorporating advanced DL architectures, hybrid models, and optimization techniques. Studies in this group have employed a combination of convolutional neural networks (CNNs), long short-term memory (LSTM), support vector machines (SVM), and vision transformers (ViT). For instance, Mahmood et al.^31^ introduced a hybrid CNN+LSTM and CNN+SVM model for the diagnosis and grading of cancerous polyps. By incorporating TL, they reduced computational time while improving classification outcomes. The integration of Grad-CAM techniques for model explainability further refined the results, achieving a sensitivity of 0.99 and an AUC of 0.99. Moreover, Boudouh and Bouakkaz^32^ proposed a dual-view architecture that simultaneously processed both cranial-caudal (CC) and mediolateral oblique (MLO) mammogram views, enhancing diagnostic sensitivity. They also introduced a hybrid ViT++ and CNN approach for breast calcification classification, achieving 96.12% accuracy, surpassing standalone models such as VGG16. Additionally, Shah et al.^33^ presented an ensemble method that integrates four state-of-the-art CNN architectures, EfficientNet, AlexNet, ResNet, and DenseNet. Each model was optimized with specific architectural modifications such as variable dropout, learnable skip connections, and selective connectivity. The ensemble achieved 94.6% accuracy, 94.6% precision, 92.4% sensitivity, 96.1% specificity, and an AUC of 98.0%, demonstrating higher diagnostic precision and stability compared to individual models. Furthermore, Almaslukh^34^ proposed a reliable deep learning framework using DenseNet-121 and random search optimization to fine-tune hyperparameters for breast cancer classification on histopathological images. The proposed model achieved an accuracy of 96.42% at 400$\times$ magnification, effectively addressing uncertainty in predictions through conformal prediction and ensuring confidence in diagnostic outcomes. Additionally, Junyue et al.^35^ proposed a hybrid AlexNet–Extreme Learning Machine (ELM) model, optimized using the Chimp Optimization Algorithm (ChOA) and the Nelder–Mead simplex (NMS) method, which addressed convergence issues and achieved high sensitivity and specificity.

A third group of researchers introduced innovative approaches to improving breast cancer detection by combining deep learning with optimization and ensemble-based machine learning techniques. For example, Sharmin et al.^36^ proposed a hybrid dependable breast cancer detection framework that integrates deep learning using a pre-trained ResNet50V2 model with ensemble-based ML methods. The DL component effectively extracted hidden patterns from complex histopathology images, while ML algorithms improved interpretability and generalization. Using the Invasive Ductal Carcinoma (IDC) dataset, the proposed hybrid system achieved 95% accuracy, 94.86% precision, 94.32% recall, and an F1-score of 94.57%, with the Light Gradient Boosting Classifier (LGB) identified as the most effective ML component. Similarly, Parshionikar and Bhattacharyya^37^ proposed a modified multi-scale CapsNet architecture optimized using the Orchard Optimization Algorithm and reported a classification accuracy of 97% on infrared thermal breast images. Kunta et al.^38^ also proposed a custom CNN model for breast cancer classification, outperforming pre-trained models such as MobileNetV3, EfficientNetB1, VGG16, and ResNet50V2, with an accuracy of 92%.

Collectively, these studies demonstrate significant advances in breast cancer detection through improved architectures, optimization, and transfer learning, enhancing efficiency and diagnostic reliability (Table 1). However, challenges persist in interpretability, robustness, and generalizability, especially in resource-limited settings, where most models remain computationally heavy or lack transparent decision-making.

**Table 1:** Summary of recent deep learning approaches for mammographic breast cancer detection and classification.

Author(s)	Methodology	Dataset(s)	Accuracy	Key contribution
Martiniussen et al.^26^	Two DL-based AI models for detection and localization	BreastScreen Norway (129,434)	–	Large-scale evaluation; >92% detection at high threshold
Rahman et al.^27^	Hybrid U-Net + YOLO model	MIAS	93%	Joint detection and localization of breast lesions
Murty et al.^28^	Hybrid CNN integrating imaging + tabular data	CBIS-DDSM, WBCD	96%	Cross-modality hybrid learning for improved adaptability
Laaffat et al.^29^	Fine-tuned MobileNetV2 model	MIAS	97%	Improved performance via TL and fine-tuning
Ruchai et al.^30^	MobileNetV2 with data augmentation	MIAS	95.8%	Demonstrated benefits of augmentation in TL
Mahmood et al.^31^	CNN+LSTM/SVM hybrid + Grad-CAM explainability	Mammographic image	–	Enhanced interpretability with high sensitivity (0.99)
Boudouh & Bouakkaz^32^	Dual-view ViT++ + CNN hybrid	CBIS-DDSM	96.12%	Multi-view processing improving diagnostic sensitivity
Shah et al.^33^	Ensemble (EfficientNet, AlexNet, ResNet, DenseNet)	RSNA	94.6%	Optimized ensemble with high precision and stability
Almaslukh^34^	DenseNet-121 + random search + conformal prediction	Histopathology (400×)	96.42%	Reliable classification with uncertainty quantification
Junyue et al.^35^	AlexNet + ELM optimized with ChOA + NMS	CBIS-DDSM, MIAS	95.32%	Hybrid optimization improving convergence
Sharmin et al.^36^	ResNet50V2 + Ensemble ML (LGB)	IDC	95%	Hybrid DL–ML model improving interpretability and generalization
Parshionikar & Bhattacharyya^37^	Multi-scale CapsNet + Orchard Optimization	Infrared Thermal	97%	Optimization-driven CapsNet achieving high performance
Kunta et al.^38^	Custom CNN model	MIAS	92%	Outperformed MobileNetV3, EfficientNetB1, VGG16, ResNet50V2

## Materials and methods

**Fig. 2:**
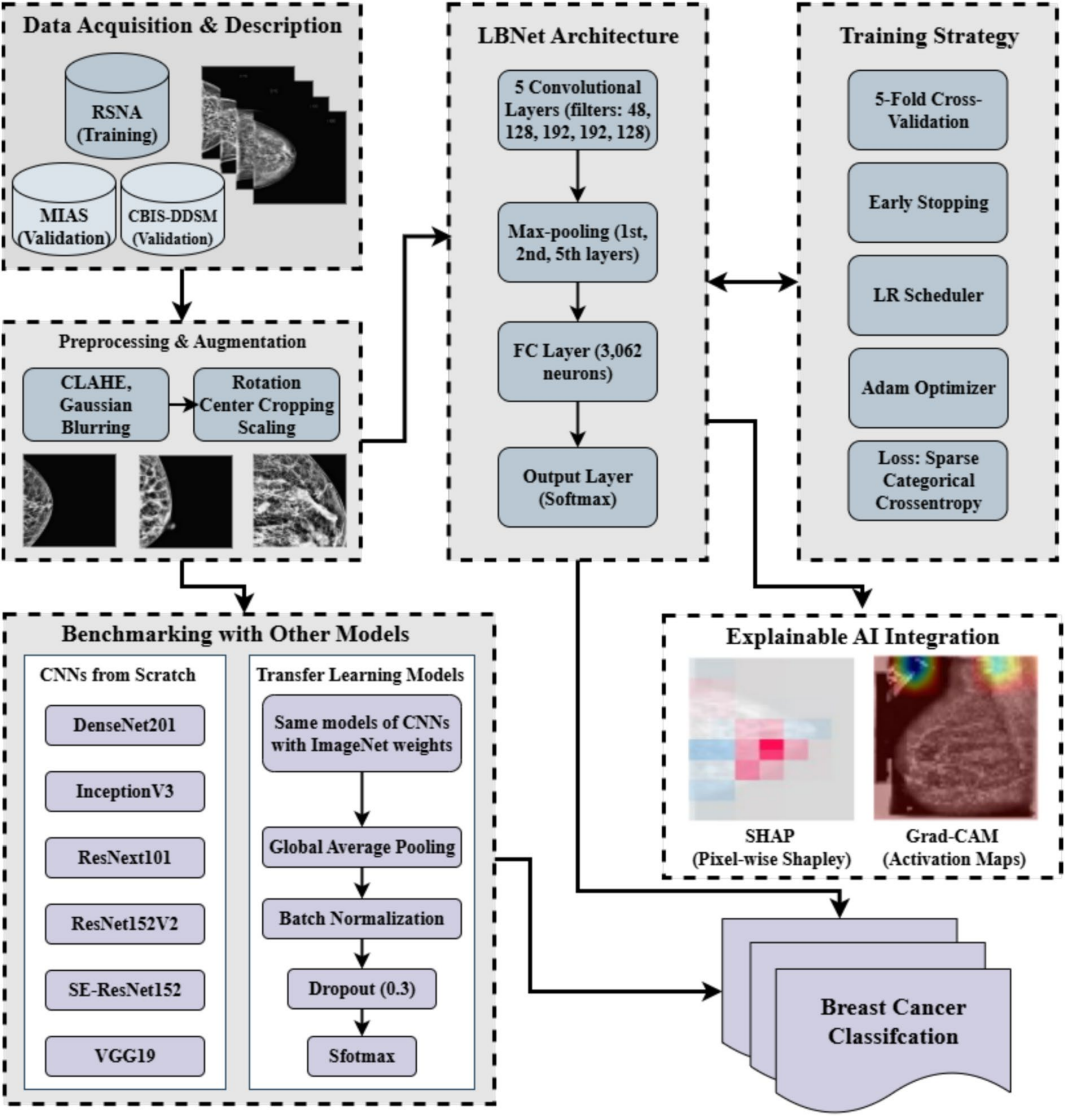
Workflow of the proposed methodology. A flowchart depicting the step-by-step process from data acquisition to benchmark comparison for breast cancer classification using LBNet.

This investigation presents a comprehensive methodology for developing and evaluating LBNet, a lightweight and interpretable CNN for classifying breast cancer using mammographic images. The approach encompasses five key stages: (i) dataset acquisition and description, (ii) preprocessing and augmentation, (iii) LBNet architecture design, (iv) integration of XAI methods, (v) benchmark comparison with state-of-the-art (SOTA) CNNs and transfer learning (TL) Models. The workflow is summarized in Fig. 2, which illustrates the process from data acquisition to model comparison. LBNet was trained on the RSNA Breast Cancer Dataset^39^ and validated on the MIAS and CBIS-DDSM datasets^40^ to assess generalizability.

### Dataset acquisition and description

**Fig. 3:**
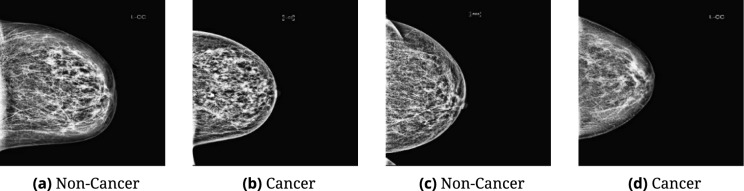
Samples of images used in the study. Representative mammography patches illustrating structural differences between non-cancerous (**a**, **c**) and cancerous (**b**, **d**) tissues across datasets.

The primary dataset used for training and internal validation is the RSNA Breast Cancer Dataset, publicly available on Kaggle^39^. It comprises 8013 grayscale mammographic images (4015 cancerous, 3998 non-cancerous) in JPEG format, each originally sized at 640 × 640 pixels. The near-balanced class distribution supports reliable binary classification. For external validation, the MIAS and CBIS-DDSM datasets from Mendeley Data^40^ were used. MIAS includes 3,816 images (1440 cancer, 2376 non-cancer), and CBIS-DDSM contains 12,328 images (7158 cancer, 5170 non-cancer). Representative examples from each dataset are illustrated in Fig. 3, visually highlighting structural differences between cancerous and non-cancerous breast tissue.

### Preprocessing pipeline and data augmentation strategy

To enhance visibility of imaging features and reduce noise, a multistep preprocessing pipeline was applied^41^. Contrast Limited Adaptive Histogram Equalization (CLAHE) was employed to improve local contrast in dense tissue regions, followed by Gaussian blurring to suppress high-frequency noise, defined as:

1 $$\begin{aligned} G(x,y) = \frac{1}{2\pi \sigma ^2} \exp \left( -\frac{x^2 + y^2}{2\sigma ^2}\right) \end{aligned}$$

where $\sigma$ represents the standard deviation of the Gaussian distribution. Subsequently, bilateral filtering was applied to preserve edge structures critical for mass detection, expressed as:

2 $$\begin{aligned} I_{\text {filtered}}(x) = \frac{1}{W_p} \sum _{x_i \in \Omega } I(x_i) \cdot f_r(|I(x_i) - I(x)|) \cdot f_s(|x_i - x|) \end{aligned}$$

where $f_r$ denotes the range kernel, $f_s$ the spatial kernel, and $W_p$ the normalization factor. Non-local means denoising was utilized to further enhance smoothness while preserving important features. Finally, unsharp masking was performed to improve edge clarity, formulated as:

3 $$\begin{aligned} I_{\text {sharp}} = I_{\text {original}} + \alpha \cdot (I_{\text {original}} - G_{\text {blur}}) \end{aligned}$$

with $\alpha = 1.5$ controlling sharpness.

To mitigate overfitting and enhance generalization, a unified set of augmentation techniques was applied across all datasets, including geometric transformations ($\pm {15}^\circ$ rotations, center cropping) and photometric adjustments (brightness/contrast modulation, random scaling). Horizontal and vertical flipping were incorporated to increase dataset diversity, as commonly practiced in mammography deep learning to simulate orientation variations^42,43^. To address concerns about anatomical distortion from flipping, a controlled experiment was conducted. LBNet was retrained without horizontal flips. Clinically aligned augmentations were retained, including $\pm {15}^\circ$ rotations and CLAHE-based contrast adjustments. Table 2 presents results indicating that excluding horizontal flips slightly reduced accuracy from 97.28% to 88.92%, with minor decreases in precision, recall, and F1-score, suggesting that flipping contributes to robustness without introducing significant label noise.

**Table 2:** Impact of horizontal flip augmentation on LBNet performance.

Setup	Accuracy (%)	Precision (%)	Recall (%)	F1-score (%)
With horizontal flip	97.28	99/96	96/99	97/97
Without horizontal flip	88.92	90/88	88/90	89/89

Metrics on the RSNA dataset with and without horizontal flips.

**Fig. 4:**
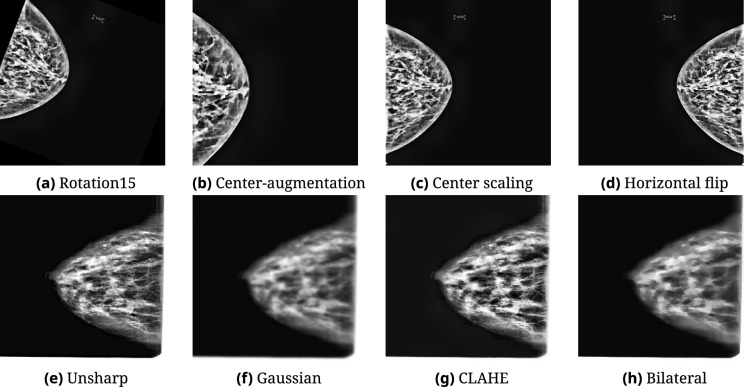
Representative data augmentations. Top row: non-cancer; bottom row: cancer. Techniques include geometric and photometric transformations as well as preprocessing filters.

Following augmentation, the RSNA dataset had 6134 cancerous and 6204 non-cancerous images. For external validation, MIAS and CBIS-DDSM were balanced to 6000 images per class (12,000 total). This was achieved via undersampling, oversampling, and the same augmentation strategies. Sample augmentation outputs are presented in Fig. 4.

### Proposed model: LBNet

The proposed LBNet is a lightweight CNN tailored for efficient and accurate breast cancer classification. It is designed with low computational cost in mind. LBNet balances depth and complexity to ensure robust performance, even in resource-constrained environments.

#### LBNet architecture

The architecture consists of five convolutional layers with progressively increasing filter sizes of 48, 128, 192, 192, and 128, respectively. Each convolutional layer is paired with a ReLU activation and batch normalization, which aids in faster convergence and ensures training stability. Max-pooling layers are applied after the first, second, and fifth convolutions to downsample the feature maps while retaining key spatial information. The spatial reductions and filter sizes produce a 512-neuron flattened vector. This provides a compact yet rich feature encoding. The vector is then fed into a dense layer with 3,062 neurons. Finally, a softmax layer with two neurons performs binary classification.

**Fig. 5:**
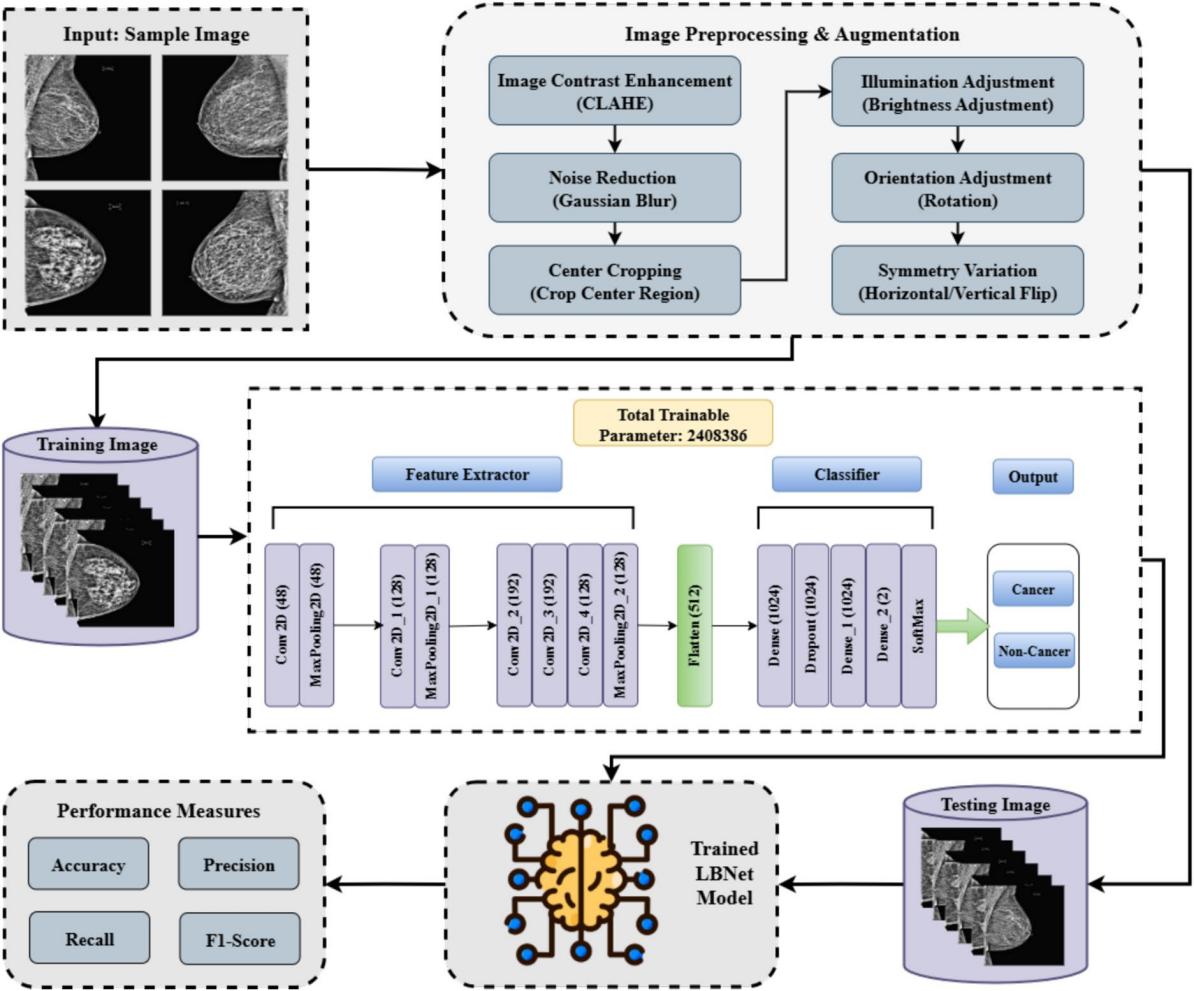
Layered architecture of LBNet. This architecture illustrates the sequential convolutional and pooling layers integrated into the lightweight LBNet model, optimized for efficient mammographic image classification.

The schematic overview of the architecture is depicted in Fig. 5.

The operations in LBNet are mathematically formalized as follows. Each convolutional layer performs:

4 $$\begin{aligned} Z^{(l)} = W^{(l)} \cdot A^{(l-1)} + b^{(l)}, \quad A^{(l)} = \text {ReLU}(Z^{(l)}) \end{aligned}$$

where $W^{(l)}$ and $b^{(l)}$ represent the weight matrix and bias vector of layer *l*, and $A^{(l-1)}$ represents the activation output from the previous layer.

Max-pooling is applied using:

5 $$\begin{aligned} A^{(l)} = \max _{(i,j) \in P_k} A_{i,j}^{(l)} \end{aligned}$$

where $P_k$ denotes the receptive field of the pooling operation.

The final prediction is generated through the softmax activation:

6 $$\begin{aligned} \hat{y} = \text {Softmax}(W_{\text {fc}} \cdot A_{\text {flatten}} + b_{\text {fc}}) \end{aligned}$$

where $\hat{y}$ is the predicted class probabilities, $\text {Softmax}(\cdot )$ maps logits to probabilities, and $W_{\text {fc}}$ is the weight matrix of the final dense layer.

Table 3 provides a comprehensive summary of each layer in the LBNet model, including output shapes and parameter counts.

**Table 3:** Architecture details of the proposed LBNet model.

Layer name	Type	Shape of output	Count of parameters
Input Layer	Input	(64, 64, 1)	–
Conv2D	Convolutional	(62, 62, 48)	1344
MaxPooling2D	Pooling	(20, 20, 48)	0
Conv2D_1	Convolutional	(20, 20, 128)	55,424
MaxPooling2D_1	Pooling	(6, 6, 128)	0
Conv2D_2	Convolutional	(6, 6, 192)	221,376
Conv2D_3	Convolutional	(6, 6, 192)	331,968
Conv2D_4	Convolutional	(6, 6, 128)	221,312
MaxPooling2D_2	Pooling	(2, 2, 128)	0
Flatten	Flatten	(512)	0
Totals	Total	–	**2,408,386**
	Trainable	–	**2,408,386**
	Non-trainable	–	**0**

Summary of each layer, including type, output shape, and parameter count.

#### Model training and optimization

LBNet was optimized using the Adam optimization method with a learning rate of $1 \times 10^{-4}$, employing sparse categorical cross-entropy as the loss function. To promote reliable generalization, five-fold cross-validation was conducted during training. An early stopping strategy with a patience of 10 epochs was implemented to prevent overfitting, and a learning rate scheduler decreased the rate by 0.1 after every 10 epochs. The complete hyperparameter configuration is summarized in Table 4.

**Table 4:** Hyperparameter tuning for LBNet.

Parameter	Value
Epochs	50
Batch size	32
Image size	(64, 64, 3)
Learning rate	1.0000e-04
Weight decay	0.0000001
K-folds	5
Optimizer	Adam (learning rate = 1.0000e-04)
Loss	Sparse Categorical Crossentropy (from_logits=True)
Early stopping	Monitor: val_accuracy, patience=10, restore best weights
Learning rate scheduler	$0.1 \times$ learning rate every 10 epochs
Callbacks	EarlyStopping, LearningRateScheduler

Parameters used for training and validating the LBNet model.

To ensure both high performance and transparency, the LBNet model is trained using a structured pipeline that incorporates cross-validation and XAI. Algorithm 1 presents the step-by-step training procedure, including model compilation, evaluation, and integration of SHAP and Grad-CAM for interpretability.

**Algorithm 1:**
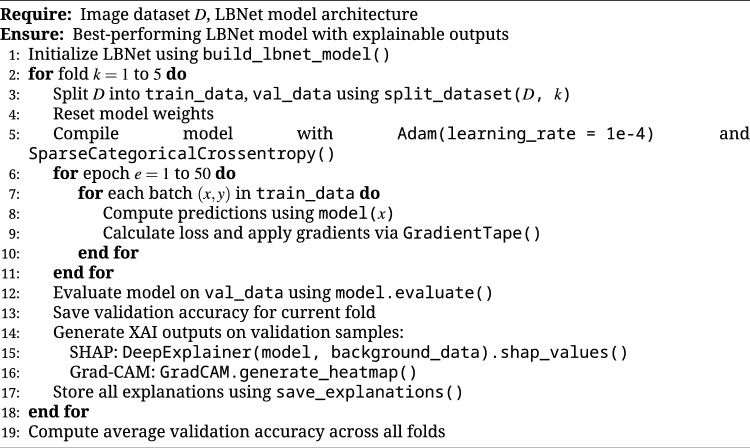
Training LBNet with XAI integration

### Explainable AI integration

To enhance clinical trust, LBNet integrates SHapley Additive exPlanations (SHAP) and Gradient-weighted Class Activation Mapping (Grad-CAM) for post-hoc explainability. SHAP computes pixel-level feature contributions using Shapley values, defined as:

7 $$\begin{aligned} \phi _i = \sum _{S \subseteq N \setminus \{i\}} \frac{|S|!(|N|-|S|-1)!}{|N|!} [f_x(S \cup \{i\}) - f_x(S)] \end{aligned}$$

where $\phi _i$ represents the contribution of feature $i$ (e.g., a pixel), $S$ denotes a subset of features excluding $i$, $N$ is the full set of features, and $f_x(S)$ is the model’s output for subset $S$. SHAP heatmaps visualize positive and negative contributions to class predictions, highlighting features that increase or decrease the likelihood of the predicted class.

Grad-CAM generates localization maps using gradients of the target class relative to the last convolutional layer’s feature maps, defined as:

8 $$\begin{aligned} \alpha _k^c = \frac{1}{Z} \sum _i \sum _j \frac{\partial y^c}{\partial A_{ij}^k} \end{aligned}$$

9 $$\begin{aligned} L_{\text {Grad-CAM}}^c = \text {ReLU}\left( \sum _k \alpha _k^c A^k\right) \end{aligned}$$

where $\alpha _k^c$ is the importance weight for feature map $k$ of class $c$, $Z$ is the total number of pixels in the feature map, $\sum _i \sum _j$ denotes global average pooling over width $i$ and height $j$, $\partial y^c / \partial A_{ij}^k$ is the gradient of the class score $y^c$ with respect to activation $A_{ij}^k$ at position $(i,j)$, $A^k$ is the feature map of the last convolutional layer, and $\text {ReLU}$ ensures non-negative heatmap values. These methods were applied to RSNA validation samples to provide visual justifications for correct and incorrect predictions.

### Benchmark models

This investigation evaluates the performance of the proposed LBNet model by comparing it against two categories of baseline models: standalone CNNs and TL-based CNNs.

**SOTA CNNs:** Six well-established convolutional neural network (CNN) architectures, VGG19, InceptionV3, ResNet152V2, DenseNet201, ResNeXt101, and SE-ResNet152, are implemented from scratch and used as benchmark models for comparative evaluation in mammographic image classification. Although developed several years ago, these models remain central to contemporary research due to their architectural stability, generalization capacity, and proven diagnostic reliability^44^. Recent studies continue to leverage these architectures for breast cancer detection: Ponraj et al.^45^ applied VGG19 in a multi-patch deep learning framework for pathology-based classification; Chelloug et al.^46^ used a modified InceptionV3 for ultrasound-based diagnosis; Ravi et al.^47^ conducted a comparative analysis of ResNet152V2 with other transfer learning models; Taifi et al.^48^ optimized DenseNet201 using refined activation functions; Nguyen et al.^49^ integrated ResNeXt101 into a multi-task learning framework; and Mashekova et al.^50^ reviewed SE-ResNet152 in multimodal imaging studies. Collectively, these investigations demonstrate that conventional scratch-trained CNN architectures remain methodologically robust and highly relevant benchmarks in recent breast cancer research.

**Transfer learning models:** The same six CNN architectures are also evaluated using transfer learning by initializing with ImageNet weights. The top layers are replaced with a custom classification head consisting of Global Average Pooling (GAP), Batch Normalization, Dropout ($p = 0.3$), and a softmax output layer. Fine-tuning is restricted to deeper layers to balance specificity and generalization, optimizing the models for mammographic classification. These architectures remain highly relevant in recent studies due to their proven performance and widespread adoption in medical imaging tasks.

###  Experimental environment setup

Experiments were performed on Google Colab with a Tesla T4 GPU, 12 GB VRAM, and 360 GB disk storage, using Python 3.10, TensorFlow 2.x, and Keras. This environment ensured rapid experimentation and consistent computational conditions.

## Results

This section presents a comprehensive analysis of the experimental results obtained through the development and evaluation of LBNet, a lightweight CNN model tailored for breast cancer classification using mammographic images. The outcomes are organized into four key parts: (i) the classification performance of LBNet using five-fold cross-validation on the RSNA dataset, (ii) validation of model generalizability on MIAS and CBIS-DDSM datasets, (iii) explainability analysis via SHAP and Grad-CAM techniques, and (iv) comparative benchmarking against state-of-the-arts (SOTA) CNNs and transfer learning (TL) models.

### LBNet performance on RSNA dataset

The LBNet model was evaluated using five-fold cross-validation on the RSNA dataset to measure its ability to classify breast cancer from mammographic images. Table 5 reports fold-wise and aggregated results, including precision, recall, specificity, F1-score, false positive rate (FPR), and support for both cancer and non-cancer classes, along with fold-wise and overall accuracy. The outcomes indicate stable learning behavior, low false positive rates, and strong discriminative power across all folds.

In Fold 1, with 1227 cancer and 1241 non-cancer cases, LBNet achieved an accuracy of 92.74%, with precision of 96% and 90%, recall of 89% and 96%, specificity of 96% and 89%, F1-scores of 92% and 93%, and FPR of 4% and 11% for cancer and non-cancer, respectively. Fold 2, comprising 1263 cancer and 1204 non-cancer cases, yielded 95.79% accuracy, precision of 98% and 94%, recall of 94% and 98%, specificity of 98% and 94%, F1-scores of 96% for both classes, and FPR of 2% and 6%. Fold 3, with 1189 cancer and 1278 non-cancer cases, reached 98.91% accuracy, precision of 99% and 98%, recall of 98% and 100%, specificity of 100% and 98%, F1-scores of 99% for both classes, and FPR of 2% and 0%. Fold 4, consisting of 1239 cancer and 1228 non-cancer cases, obtained 99.19% accuracy, precision of 100% and 99%, recall of 99% and 100%, specificity of 100% and 99%, F1-scores of 99% for both classes, and FPR of 1% and 0%. Fold 5, with 1216 cancer and 1251 non-cancer cases, achieved 99.84% accuracy, with perfect precision, recall, specificity, F1-scores, and FPR (100% / 0%) for both classes.

Aggregated across all folds, LBNet achieved an overall accuracy of 97.28%, with precision of 99% for cancer and 96% for non-cancer, recall of 96% and 99%, specificity of 99% and 96%, F1-scores of 97% for both classes, and FPR of 1% and 4%, based on 6134 cancer and 6204 non-cancer cases. These results show that LBNet consistently distinguishes cancer from non-cancer. It achieves high reliability, low false positive rates, and strong generalization on the RSNA dataset.

**Table 5:** Five-fold cross-validation and aggregated performance of LBNet on the RSNA dataset.

Fold	Metric	Cancer	Non-Cancer	Accuracy
Fold 1	Precision	96%	90%	**92.74%**
	Recall	89%	96%	
	Specificity	96%	89%	
	F1-score	92%	93%	
	FPR	4%	11%	
	Support (N)	1227	1241	
Fold 2	Precision	98%	94%	**95.79%**
	Recall	94%	98%	
	Specificity	98%	94%	
	F1-score	96%	96%	
	FPR	2%	6%	
	Support (N)	1263	1204	
Fold 3	Precision	99%	98%	**98.91%**
	Recall	98%	100%	
	Specificity	100%	98%	
	F1-score	99%	99%	
	FPR	2%	0%	
	Support (N)	1189	1278	
Fold 4	Precision	100%	99%	**99.19%**
	Recall	99%	100%	
	Specificity	100%	99%	
	F1-score	99%	99%	
	FPR	1%	0%	
	Support (N)	1239	1228	
Fold 5	Precision	100%	100%	**99.84%**
	Recall	100%	100%	
	Specificity	100%	100%	
	F1-score	100%	100%	
	FPR	0%	0%	
	Support (N)	1216	1251	
Aggregated	Precision	99%	96%	**97.28%**
	Recall	96%	99%	
	Specificity	99%	96%	
	F1-score	97%	97%	
	FPR	1%	4%	
	Support (N)	6134	6204	

Metrics include precision, recall, specificity, F1-score, and false positive rate (FPR) for cancer and non-cancer classes, along with fold-wise and overall accuracy.

Fold-wise results and aggregated metrics confirm LBNet’s high and stable classification performance across precision, recall, specificity, and low false positive rates, demonstrating strong generalization on the RSNA dataset

**Fig. 6:**
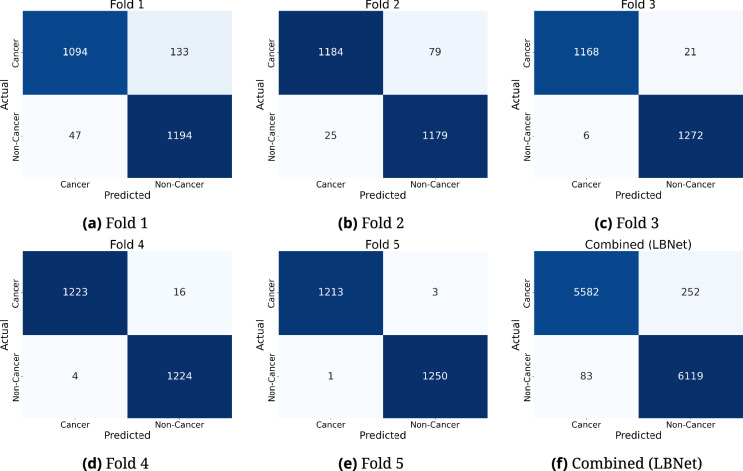
Five-fold and combined confusion matrices of LBNet. The fold-wise matrices (**a**–**e**) illustrate progressive improvement in classification accuracy, with reduced false positives and negatives. The combined matrix (**f**) summarizes overall performance.

Figure 6 shows the confusion matrices for each fold (a–e) and the combined dataset (f). Fold-wise matrices highlight reduced false positives and false negatives over successive folds, with Fold 5 approaching perfect classification. The combined matrix validates the aggregated results, with LBNet correctly classifying 5882 of 6134 cancer cases and 6119 of 6204 non-cancer cases.

**Fig. 7:**
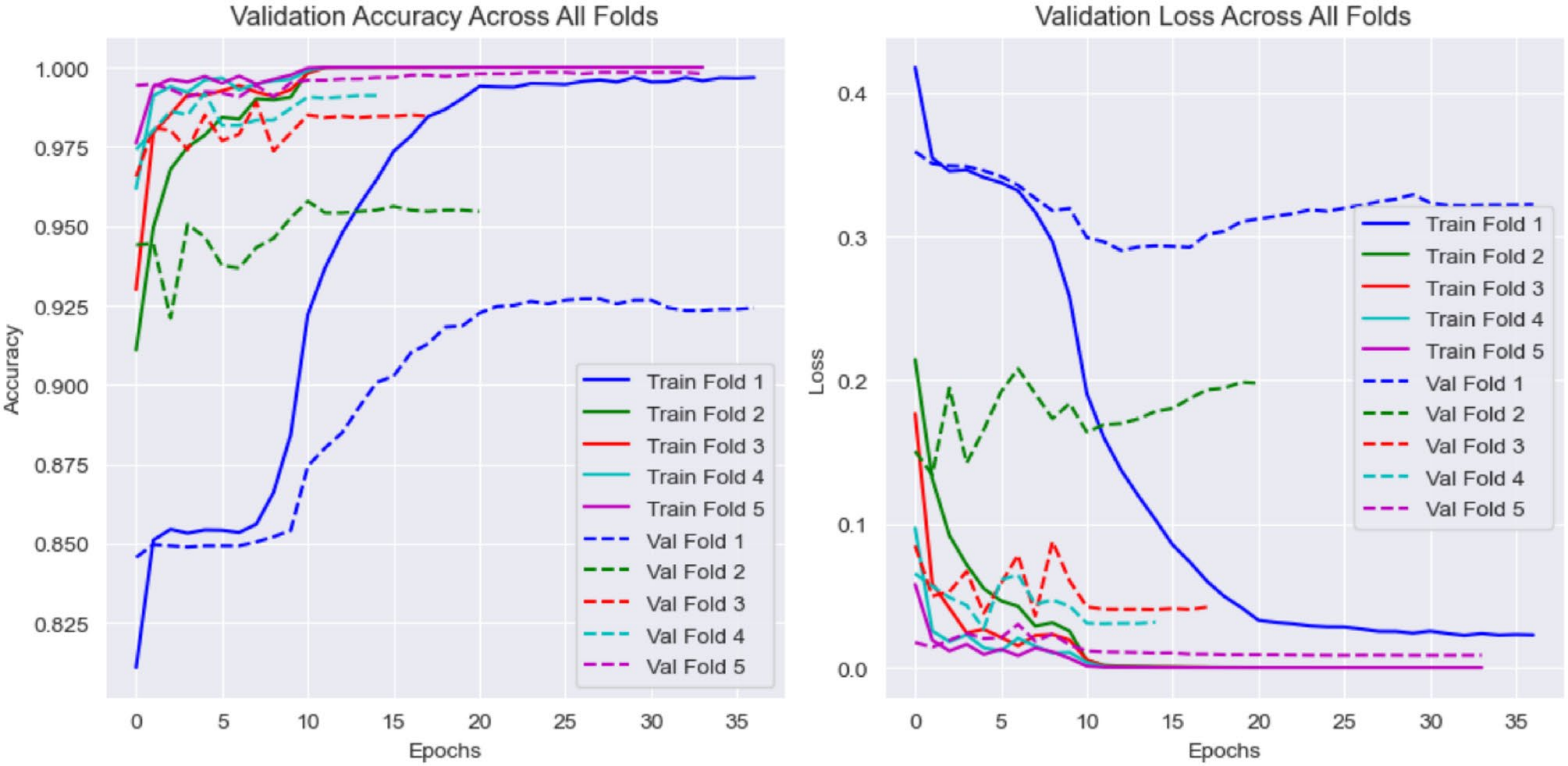
Validation accuracy and loss of all folds..

Model convergence across folds is depicted in Fig. 7, showing training and validation performance over 40 epochs. Validation accuracy increased steadily during the first 10 epochs, stabilizing above 90% and approaching 100% by epoch 30. Simultaneously, validation loss decreased and plateaued, reflecting minimal overfitting and effective learning.

### LBNet validation on MIAS and CBIS-DDSM datasets

To validate LBNet’s generalization ability beyond RSNA, the trained model was applied to the MIAS and CBIS-DDSM datasets. As presented in Table 6, LBNet demonstrated strong performance in ensuring generalizability.

**Table 6:** Classification report of LBNet on CBIS-DDSM and MIAS datasets.

Dataset	Metric	Cancer	Non-cancer	Accuracy
CBIS-DDSM	Precision	99.7%	99.4%	**99.54%**
	Recall	99.4%	99.7%	
	Specificity	99.7%	99.4%	
	F1-score	99.5%	99.5%	
	FPR	0.3%	0.6%	
	Support (N)	1200	1200	
MIAS	Precision	98.4%	98.6%	**98.50%**
	Recall	98.6%	98.4%	
	Specificity	98.4%	98.6%	
	F1-score	98.5%	98.5%	
	FPR	1.6%	1.4%	
	Support (N)	1200	1200	

External validation metrics include precision, recall, specificity, F1-score, and false positive rate (FPR).

LBNet maintains excellent external generalization with consistently high precision, recall, and specificity, while achieving very low false positive rates on both CBIS-DDSM and MIAS datasets.

Significant values are in [bold].

On the CBIS-DDSM dataset, LBNet achieved an exceptional accuracy of 99.54%. Precision was 99.7% for cancer and 99.4% for non-cancer, while recall values were 99.4% and 99.7%, respectively, resulting in F1-scores of 99.5% for both classes. The false positive rates were extremely low, at 0.3% for cancer and 0.6% for non-cancer, with only 34 misclassified instances out of 2,400, demonstrating robust cross-dataset transferability. Similarly, on the MIAS dataset, LBNet maintained an impressive accuracy of 98.50%. Precision was 98.4% for cancer and 98.6% for non-cancer, while recall values were 98.6% and 98.4%, yielding F1-scores of 98.5% for both classes. False positive rates were low, at 1.6% for cancer and 1.4% for non-cancer, confirming stable performance across datasets. These results highlight LBNet’s strong external generalization. It adapts well to diverse imaging protocols. This supports its potential for reliable deployment in varied clinical settings.

### Explainability with SHAP and Grad-CAM

To enhance clinical transparency, SHapley Additive exPlanations (SHAP) and Gradient-weighted Class Activation Mapping (Grad-CAM) were applied for post-hoc interpretability of LBNet’s predictions, highlighting diagnostically relevant features.

**Fig. 8:**
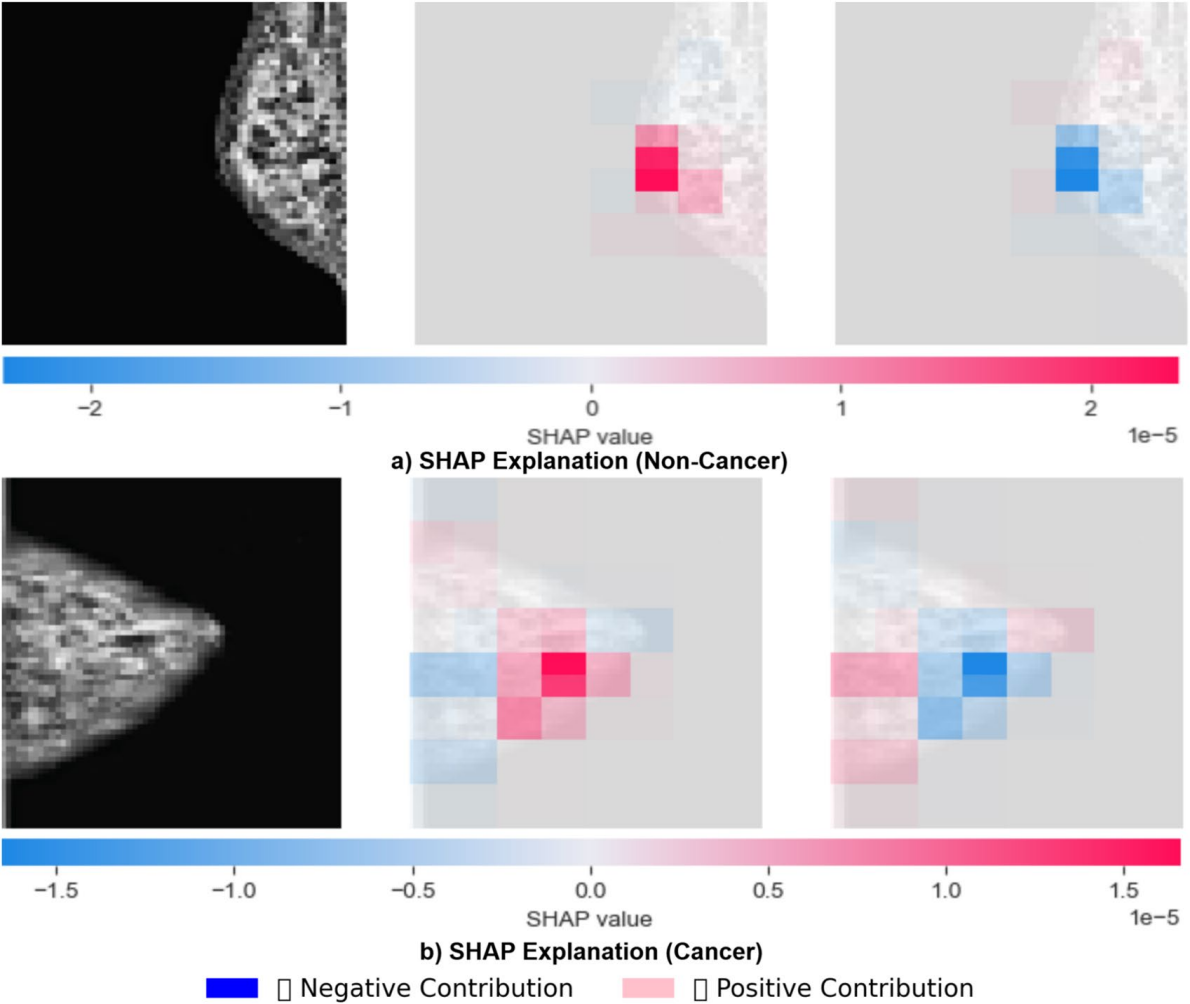
SHAP visualization. Pixel contributions for (**a**) non-cancer and (**b**) cancer cases. Red increases predicted class likelihood, blue supports the alternative, with intensity showing magnitude.

SHAP assigns pixel-level contributions relative to a baseline, visualized in Fig. 8, where red regions indicate positive contributions toward the predicted class (e.g., cancer) and blue regions support the negative contributions (e.g., non-cancer). Model predictions arise from the net balance of these contributions. For instance, Fig. 8(a) (non-cancer) displays predominant blue activation for normal tissue, while Fig. 8(b) (cancer) highlights red regions corresponding to masses. The intensity of each color reflects the magnitude of contribution.

**Fig. 9:**
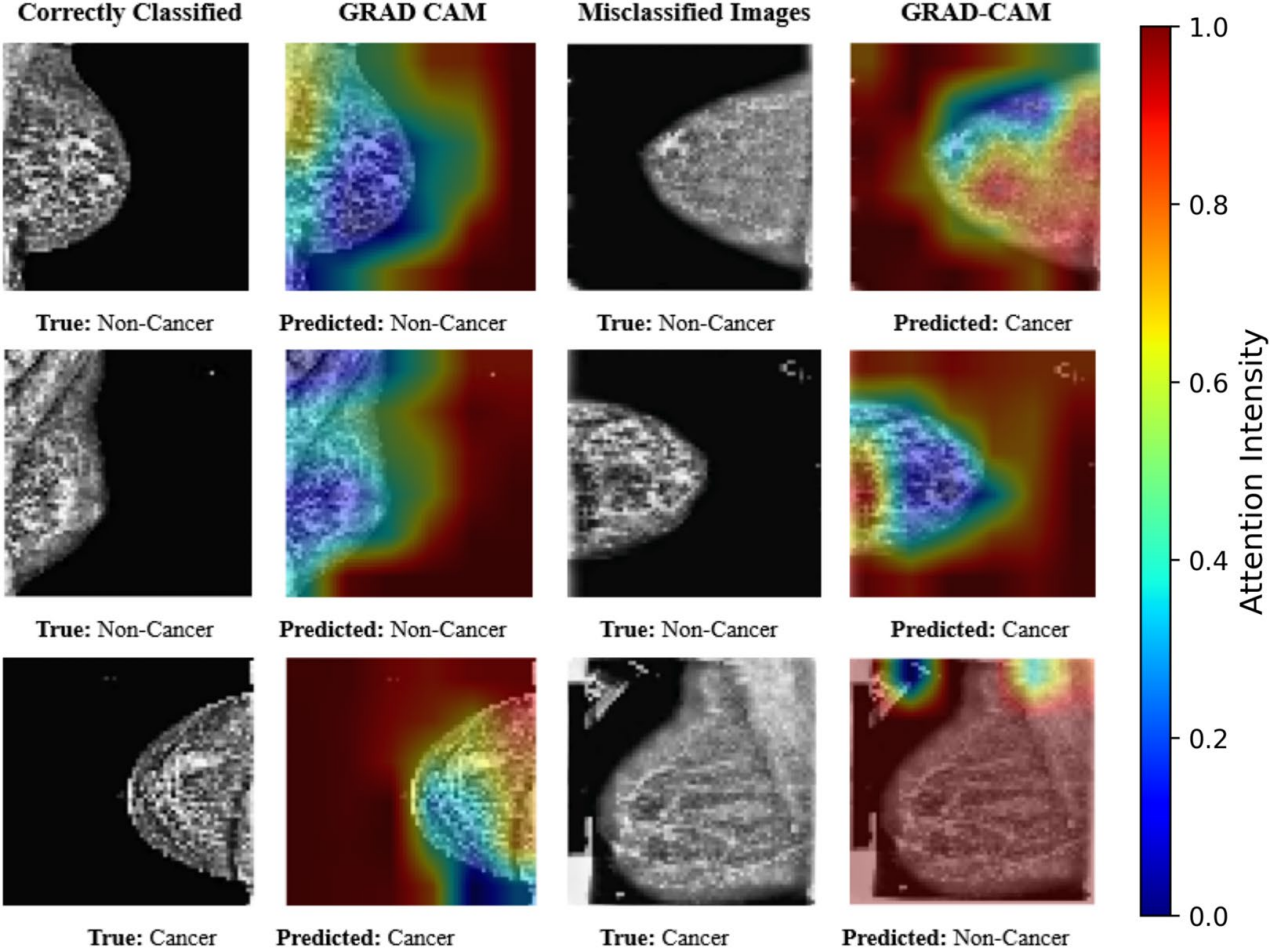
Grad-CAM visualization. Red denotes high attention, blue low.

Grad-CAM was applied to 1000 RSNA images to visualize spatial attention. In line with LBNet’s overall accuracy of 97.28%, the vast majority of cases exhibited attention maps focusing on clinically meaningful regions, red indicating high relevance (e.g., masses in cancer) and blue denoting low attention (e.g., normal tissue in non-cancer). In the minority of cases corresponding to misclassifications, Grad-CAM maps typically focused on ambiguous or atypical areas. The color bars indicate attention intensity (red: high to blue: low). While specific class-wise counts were not recorded for these 1000 images, the proportion of correctly classified cases mirrors the overall model performance. (Fig. 9)

**Clinical Alignment and Reliability.** SHAP and Grad-CAM collectively demonstrate that LBNet emphasizes relevant features, with pixel-level and spatial attention maps aligning with expected breast tissue patterns. The high proportion of correctly classified cases, as reflected in the overall precision of the model, provides confidence in the interpretability of LBNet predictions. Misclassified cases reveal attention patterns predominantly in ambiguous regions, which supports a transparent understanding of model errors. This combined qualitative and proportional evaluation substantiates the clinical alignment and usability of LBNet’s interpretability outputs.

### Benchmark comparison

**Fig. 10:**
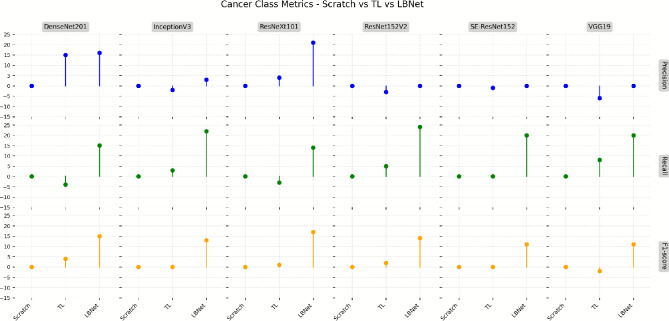
Comparison of cancer class metrics for scratch vs. TL models. The lollipop chart illustrates differences in precision, recall, and F1-score for cancer cases across baseline models on the RSNA dataset.

This investigation evaluated the performance of six baseline CNN architectures, DenseNet201, InceptionV3, ResNeXt101, ResNet152V2, SE-ResNet152, and VGG19, on the RSNA dataset, using both scratch training and transfer learning with ImageNet weights. Fig. 10 presents a lollipop chart comparing the performance of these models on the cancer class, highlighting the limitations of scratch-trained variants. Transfer learning implementations, including ResNet152V2 and DenseNet201, exhibited recall improvements of 6.2% for ResNet152V2 and 6.8% for DenseNet201 over their scratch-trained counterparts. LBNet further advanced this metric, achieving a recall improvement of 26.3% compared to the average of scratch-trained baseline models, underscoring its superior sensitivity in detecting cancer masses and microcalcifications.

**Fig. 11:**
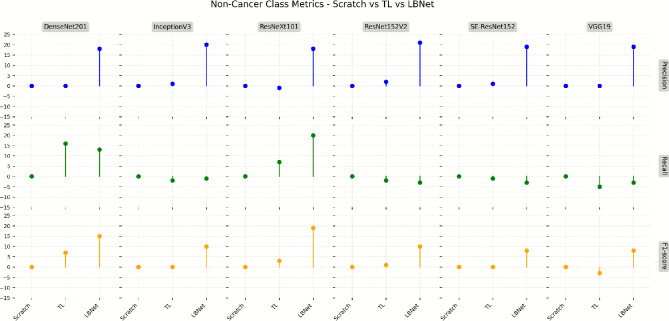
Comparison of non-cancer class metrics for scratch vs. TL models. The lollipop chart depicts differences in precision, recall, and F1-score for non-cancer cases across baseline models on the RSNA dataset.

Fig. 11 compares the performance on non-cancer cases. Scratch-trained models, including VGG19 and SE-ResNet152, achieved a recall of 99% each. With transfer learning, ResNet152V2 and DenseNet201 improved precision by 2.4% and 3.1%, respectively, reducing false positives. LBNet outperformed all models, increasing precision by 18.7% compared to the average of scratch-trained models, ensuring accurate identification of benign tissue while maintaining high recall, thus addressing class imbalance issues.

**Fig. 12:**
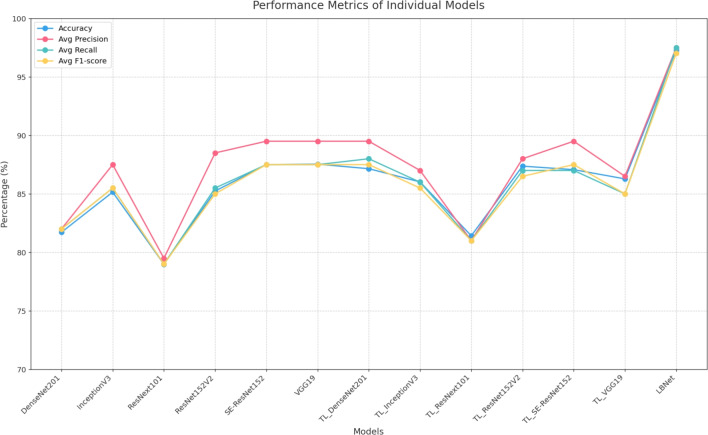
Performance comparison of LBNet with baseline models..

Fig. 12 shows that LBNet achieved an overall accuracy of 97.28%, which is 11.16% higher than VGG19 (87.54% from scratch) and 11.40% higher than ResNet152V2 (87.37% using transfer learning).For cancer classification, LBNet achieves 99% precision, 96% recall, and 97% F1-score. It surpasses ResNet152V2 with a 3.13% precision improvement. It also shows a 24.68% recall enhancement and a 14.12% F1-score boost. For non-cancer cases, LBNet achieves 96% precision, 99% recall, and 97% F1-score. It outpaces VGG19 (scratch) with a 20.00% precision gain. It maintains 99% recall and shows a 9.89% F1-score improvement.

## Discussion

**Fig. 13:**
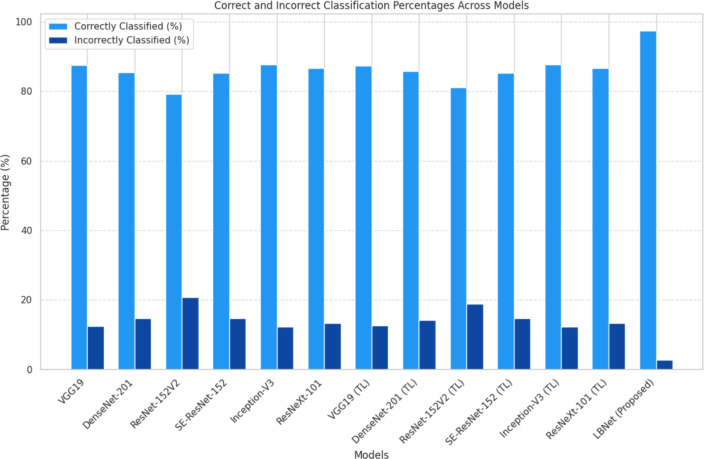
Comparison of classified and misclassified instances across models. LBNet achieves the best classification rate (97.02%) and lowest error (2.98%), surpassing all baselines.

This study demonstrates LBNet’s superior performance in breast cancer detection, achieving 97.28% accuracy on the RSNA dataset and surpassing baseline models such as VGG19 (87.54%), SE-ResNet152 (87.50%), and ResNet152V2 (85.24%) trained from scratch, as well as ResNet152V2 (87.37%) and DenseNet201 (87.15%) under transfer learning. This success stems from LBNet’s compact five-layer architecture (2.4M parameters). It uses progressive filter growth (48$\rightarrow$128$\rightarrow$192$\rightarrow$192 $\rightarrow$128), ReLU activations, and batch normalization. These ensure efficient feature extraction and training stability. Combined with five-fold cross-validation and Adam optimization, LBNet uses domain-aligned preprocessing. This includes CLAHE, Gaussian blur, and unsharp masking. Balanced augmentation ($\pm {15}^\circ$ rotation, intensity shifts) is also applied. As a result, LBNet achieves 99% precision in cancer cases and 99% recall in non-cancer cases. It maintains specificity above 98% with FPR below 2%. This yields gains of up to 12.04% in accuracy and 26.3% in cancer recall over baselines. Remarkably, it uses only 1/8th to 1/27th the parameters. Fig. 13 highlights LBNet’s highest correct classification rate (97.02%). It also shows the lowest misclassification rate (2.98%). In comparison, VGG19 (Scratch) achieves 86.17% correct and 13.83% incorrect. DenseNet201 (TL) shows 83.02% correct and 16.98% incorrect. This demonstrates LBNet’s strong reliability and diagnostic robustness across folds.

A major strength of LBNet lies in computational efficiency, making it practical for clinical deployment. With only 2.4M parameters, it is substantially lighter than InceptionV3 (21.8M), DenseNet201 (18.3M), ResNext101 (42.3M), ResNet152V2 (58.3M), SeResNet152 (64.9M), and VGG19 (20.0M), as summarized in Table 7. Efficiency was further validated through latency, FLOPs, and memory analysis. Metrics were derived from TPU training logs with $256 \times 256 \times 3$ inputs and batch size 32. Inference latency was estimated from per-step training times, FLOPs from parameter count and architectural depth, and memory usage from parameter storage plus activations (training memory = 1.5$\times$ inference memory). LBNet achieves the lowest latency (23.33 ms/image) compared to SeResNet152 (241.38 ms) and ResNext101 (844.83 ms). Its FLOPs (7.92) and memory usage (11.31 MB inference, 16.97 MB training) remain minimal relative to baseline ranges (131.77–639.79 GFLOPs, 83.12–279.45 MB).

**Table 7:** Computational efficiency of LBNet vs. baseline CNNs.

Model	Params (M)	Latency (ms/img)	GFLOPs	Inf. Mem. (MB)	Train Mem. (MB)
DenseNet201	18.33	252.64	180.76	104.86	157.29
InceptionV3	21.81	96.58	171.78	97.84	146.76
ResNext101	42.27	844.83	388.89	182.84	274.26
ResNet152V2	58.34	193.10	497.70	245.67	368.51
SeResNet152	64.93	241.38	639.79	279.45	419.18
VGG19	20.03	136.78	131.77	83.12	124.68
LBNet (Proposed)	**2.40**	**23.33**	**7.92**	**11.31**	**16.97**

Metrics from TPU logs with batch size = 32.

Significant values are in [bold].

These findings confirm that LBNet is not only compact but also excels in speed, FLOPs, and memory efficiency, making it ideal for real-time clinical use. LBNet also exhibited strong generalizability across external datasets, achieving 99.54% on CBIS-DDSM and 98.50% on MIAS. This consistency addresses the limitation of CNNs that often underperform across heterogeneous datasets. Compared with recent literature (Table 8), the proposed LBNet model demonstrated superior diagnostic performance. Specifically, it outperformed MobileNetV2 (95.8%, Ruchai et al.^30^), fine-tuned MobileNetV2 (97%, Laaffat et al.^29^), ViT–CNN hybrids (96.12%, Boudouh & Bouakkaz^32^), DenseNet121 with conformal prediction (96.42%, Almaslukh^34^), and the multi-scale CapsNet (97%, Parshionikar & Bhattacharyya^37^). Furthermore, it surpassed ensemble CNN architectures (94.6%, Shah et al.^33^). It also outperformed hybrid DL–ML systems (95%, Sharmin et al.^36^), confirming LBNet’s superior diagnostic generalization across diverse mammographic datasets.

**Table 8:** Performance comparison of LBNet with existing deep learning models for breast cancer classification.

Author(s)	Mode/methodology	Dataset(s)	Accuracy
Ruchai et al.^30^	MobileNetV2 + data augmentation	Digital Mammography	95.8%
Laaffat et al.^29^	Fine-tuned MobileNetV2	MIAS	97%
Junyue et al.^35^	AlexNet + ELM optimized with ChOA + NMS	CBIS-DDSM, MIAS	95.32%
Boudouh & Bouakkaz^32^	Dual-view ViT++ + CNN hybrid	CBIS-DDSM	96.12%
Almaslukh^34^	DenseNet-121 + random search + conformal prediction	Histopathology (400$\times$)	96.42%
Sharmin et al.^36^	ResNet50V2 + Ensemble ML (LGB)	IDC	95%
Shah et al.^33^	Ensemble (EfficientNet, AlexNet, ResNet, DenseNet)	RSNA	94.6%
Murty et al.^28^	CNN hybrid (tabular + imaging)	CBIS-DDSM, WBCD	96%
Parshionikar & Bhattacharyya^37^	Multi-scale CapsNet + Orchard Optimization	Infrared thermal	97%
Kunta et al.^38^	Custom CNN	MIAS	92%
This study	LBNet (Proposed)	**RSNA**	**97.28%**
		**CBIS-DDSM**	**99.54%**
		**MIAS**	**98.50%**

Significant values are in [bold].

While SHAP and Grad-CAM enhance interpretability, post-hoc XAI methods have limitations. SHAP is computationally complex and sensitive to input perturbations. This can affect feature attribution reliability in noisy mammograms. Similarly, Grad-CAM has coarse resolution. It may highlight broader, less clinically specific regions. These challenges underscore the need for future work on inherently interpretable architectures and quantitative validation to further strengthen clinical applicability.

## Conclusion

The global increase in breast cancer cases emphasizes the urgent need for diagnostic tools that are accurate, efficient, and interpretable to enable early detection and improve patient outcomes. LBNet addresses these demands with its lightweight architecture, integrating SHAP and Grad-CAM for explainable predictions, rapid inference, and minimal memory usage. Unlike traditional CNNs, LBNet requires fewer computational resources. It also handles heterogeneous imaging datasets more effectively. This makes it a scalable solution for resource-constrained healthcare settings. It outperforms larger models like ResNet152V2, SeResNet152, and DenseNet201, achieving state-of-the-art performance with lower latency, fewer FLOPs, and a reduced memory footprint. Its robust generalizability across diverse datasets further highlights its potential for real-time clinical decision support, overcoming limitations of conventional CNNs in adapting to varied imaging conditions.

Despite its strengths, LBNet has limitations that require further exploration. Currently validated only on grayscale mammograms, its performance on multimodal imaging, such as ultrasound, MRI, or temporal mammography, remains untested. Additionally, its interpretability lacks quantitative validation against radiologist-annotated regions of interest (ROI), which is critical for clinical trust. Future efforts should extend LBNet to multimodal imaging. They should also conduct quantitative XAI validation using ROI-overlap metrics with radiologists. Finally, prospective, multi-center clinical trials are needed to evaluate real-world reliability. These advancements will enhance LBNet’s interpretability, scalability, and readiness for clinical integration. They position LBNet as a transformative tool for breast cancer screening and patient care.

## Data Availability

The datasets used in this investigation are publicly available. The RSNA Breast Cancer Dataset is accessible via Kaggle^39^, while the MIAS and CBIS-DDSM datasets are available through Mendeley Data^40^.
